# Increased striatal functional connectivity with auditory cortex in tinnitus

**DOI:** 10.3389/fnhum.2015.00568

**Published:** 2015-10-28

**Authors:** Leighton B. Hinkley, Danielle Mizuiri, OiSaeng Hong, Srikantan S. Nagarajan, Steven W. Cheung

**Affiliations:** ^1^Department of Radiology and Biomedical Imaging, University of California at San Francisco, San FranciscoCA, USA; ^2^Department of Community Health Systems, School of Nursing, University of California at San Francisco, San FranciscoCA, USA; ^3^Department of Otolaryngology-Head and Neck Surgery, University of California at San Francisco, San FranciscoCA, USA; ^4^Surgical Services, San Francisco Veterans Affairs Medical Center, San FranciscoCA, USA

**Keywords:** resting-state fMRI, tinnitus, striatum, auditory cortex, functional connectivity

## Abstract

Tinnitus is a common auditory perceptual disorder whose neural substrates are under intense debate. One physiologically based model posits the dorsal striatum to play a key role in gating auditory phantoms to perceptual awareness. Here, we directly test this model along with the roles of auditory and auditory-limbic networks in tinnitus non-invasively by comparing resting-state fMRI functional connectivity patterns in chronic tinnitus patients against matched control subjects without hearing loss. We assess resting-state functional connectivity of the caudate dorsal striatum (area LC), caudate head (CH), nucleus accumbens (NA), and primary auditory cortex (A1) to determine patterns of abnormal connectivity. In chronic tinnitus, increases in ipsilateral striatal–auditory cortical connectivity are found consistently only in area LC. Other patterns of increased connectivity are as follows: (1) right striatal area LC, A1, CH, and NA with parietal cortex, (2) left and right CHs with dorsal pre-frontal cortex, (3) NA and A1 with cerebellum, hippocampus, visual and ventral pre-frontal cortex. Those findings provide further support for a striatal gating model of tinnitus, where dysfunctionally permissive area LC enables auditory phantoms to reach perceptual awareness.

## Introduction

Tinnitus is a common perceptual disorder of auditory phantoms where peripheral audiometric hearing loss (HL) profiles alone cannot help clinicians to distinguish between patients who merely experience tinnitus from those who suffer from tinnitus (Coles, 1984; Tsai et al., 2012). Central auditory system hypotheses of tinnitus genesis have been proposed to account for the discrepancy between audiometric profiles and tinnitus perceptual attributes, including lemniscal system hyperactivity (Chen and Jastreboff, 1995; Norena and Eggermont, 2003; Kaltenbach, 2006), tonotopic map plasticity (Komiya and Eggermont, 2000; Syka, 2002; Roberts et al., 2010) and thalamocortical dysrhythmia (Llinas et al., 1999; Weisz et al., 2007) in frequencies including gamma (van der Loo et al., 2009; De Ridder et al., 2011). While those oscillatory (Sedley et al., 2012) and network (Husain, 2007) state models may ultimately prove to be requisite neurophysiological substrates underlying tinnitus, they do not address mechanisms of tinnitus awareness.

A recent development is the striatal gating model (Larson and Cheung, 2012), which hypothesizes the caudate nucleus to act as a gating mechanism for tinnitus awareness. The striatal gating model is physiologically based, motivated by electrical stimulation experiments in dorsal striatal area LC, located at the junction of the head and body of the caudate nucleus, on awake and interactive humans. Direct stimulation of area LC during deep brain stimulation (DBS) surgery in movement disorders patients with comorbid chronic tinnitus modulates auditory phantom loudness (Cheung and Larson, 2010) and triggers auditory phantom percepts in HL patients without tinnitus (Larson and Cheung, 2012). Furthermore, vascular infarction of area LC results in enduring tinnitus loudness suppression (Larson and Cheung, 2013). According to this model, dysfunctional corticostriatal connections between the dorsal striatum and auditory cortex act as a pathway for auditory phantom representations to reach perceptual awareness. The normally restrictive dorsal striatum becomes pathologically permissive in chronic tinnitus. Although the physiological mechanisms are not clear, it has been proposed that alteration in the balance of excitation and inhibition either within the caudate or in its connections to auditory cortex modulates this permissiveness (Calabresi et al., 2000; Goubard et al., 2011). The striatal gating model is complementary to other central nervous system hypotheses, including those that posit tinnitus is primarily an expectation mismatch within the auditory system (primary auditory cortex (A1); Eggermont and Roberts, 2004; Roberts et al., 2013) or is driven by abnormal auditory-limbic interactions [i.e., nucleus accumbens (NA); Leaver et al., 2011; Seydell-Greenwald et al., 2012]. While invasive direct electrical stimulation studies of the dorsal striatum in movement disorder patients with comorbid tinnitus provide support for a causal role of the basal ganglia in auditory phantom perception, to date no non-invasive neuroimaging study has directly tested the physiologically based striatal gating model in the more common subpopulation of chronic tinnitus patients without movement disorder.

Here, we test the tinnitus striatal gating model directly using seeded coherence of resting-state fMRI on a cohort of chronic, constant tinnitus patients accounted for HL and a cohort of matched control individuals without tinnitus and without HL. Prior non-invasive studies of neuroanatomical connectivity and task-induced activation in the brain have provided evidence for alterations in neural structure and function in tinnitus, but only a handful have examined coherent activity in the brain at resting-state using EEG or fMRI (Vanneste et al., 2011; Kim et al., 2012; Maudoux et al., 2012a,b, Schmidt et al., 2013, Chen et al., 2014, 2015; Davies et al., 2014; Husain and Schmidt, 2014; Vanneste and De Ridder, 2015). None have directly assessed what roles striatal sub-divisions may play in auditory phantom perception. If area LC plays a prominent role in blocking phantom percepts and this mechanism breaks down in tinnitus, then alterations in resting-state functional connectivity should be observed between this structure and auditory cortical fields. Changes in resting-state connectivity for dorsal striatal area LC should be functionally distinct from other striatal sub-divisions and cortical structures involved in auditory perception. We hypothesize that in chronic tinnitus, abnormal functional connectivity between area LC and auditory cortices will be distinct from neighboring fields in the striatum.

## Materials and Methods

### Participants

Fifteen patients (**Table 1**) with chronic, constant tinnitus (TIN) aged 30–63 years (*M*_age_ = 53.5 years, *SD* = 13; 3 females) were recruited from Otolaryngology and Audiology clinics affiliated with the University of California, San Francisco (UCSF). All patients underwent standard clinical audiometry to measure pure tone thresholds and completed the Tinnitus Handicap Inventory (THI; Newman et al., 1996) to assess tinnitus severity. Pure tone audiometric thresholds for low (0.5, 1.0, and 2.0 kHz) and high (4.0, 6.0, and 8.0 kHz) frequency bands were averaged separately to assess HL degree. HL rating was determined by the poorer ear, irrespective of frequency band. The Clark (1981) HL degree scale was adapted to construct the following rating measure: 1 (–10–25 dB), 2 (26–40 dB), 3 (41–55), 4 (56–70 dB), 5 (71–90 dB), and 6 (90+ dB). This metric was used as a covariate in subsequent analysis to account for variability in HL levels within the chronic tinnitus cohort. In addition, 15 healthy control (CON) participants without tinnitus or HL (HL = 1) were recruited from the greater San Francisco Bay Area, matched for age (*M*_age_=57 years, *SD* = 12) and gender, but not for HL. All participants gave written informed consent following explanation of study procedures that were approved by the UCSF Committee on Human Research. All experiments were conducted in accordance with the Declaration of Helsinki.

**Table 1:** Demographic descriptions of subjects with chronic tinnitus.

Audiometric data										
SID	Age	Gender	Handed	Left low	Left high	Right low	Right high	Rating	Tinnitus localization	THI
TIN 001	45	Male	Right	28	80	30	80	5	Right and left ears	78
TIN 002	40	Male	Right	10	21	3	23	1	Right and left ears	48
TIN 003	41	Female	Right	48	52	20	8	3	Left ear	14
TIN 004	29	Male	Right	5	8	48	48	3	Right ear	34
TIN 005	63	Male	Right	18	27	22	40	2	Right and left ears	18
TIN 006	69	Male	Right	40	68	30	67	4	Right and left ears	36
TIN 007	45	Female	Right	10	38	3	60	4	Right ear	35
TIN 008	40	Female	Right	22	12	73	50	5	Right ear	36
TIN 009	66	Male	Right	28	83	20	72	5	Right and left ears	52
TIN 010	67	Male	Right	38	60	28	60	4	Right and left ears	32
TIN 011	69	Male	Right	27	50	25	45	3	Right and left ears	44
TIN 012	*55*	Male	Right	8	20	8	23	1	Right and left ears	86
TIN 013	52	Male	Right	7	40	7	30	2	Left ear	22
TIN 014	63	Male	Right	12	42	12	37	3	Right and left ears	12
TIN 015	37	Male	Right	67	72	8	12	5	Left ear	38

*Descriptors include age, gender, handedness, hearing profiles in the low and high frequency bands for the left and right ears, HL rating of the poorer frequency band and ear, location of tinnitus, and Tinnitus Handicap Inventory (THI) scores. HL rating (Rating): 1, normal to slight; 2, mild; 3, moderate loss; 4, moderately severe loss; 5, severe loss; 6, profound loss.*

### MRI Acquisition

MRI data was acquired using a 3.0T Siemens Trio (Siemens, Erlangen, Germany) at the UCSF Neuroscience Imaging Center (NIC). For each subject, a high-resolution anatomical MRI was acquired (MPRAGE; 160 1 mm slices, FOV = 256 mm, TR = 2300 ms, TE = 2.98 ms). Eight minutes (240 repetitions) of spontaneous fMRI data was collected (supine position, eyes closed) with a gradient echo-planar imaging (EPI) sequence (38 3.0 mm × 3.0 mm × 3.0 mm slides, TR = 2000 ms, TE = 28 ms).

### Data Preprocessing

Resting-state fMRI data was spatially pre-processed and EPI images were spatially realigned to a mean image and coregistered with the T1 image for each individual subject using SPM8^1^. All T1 images were segmented into gray and white matter images and spatially normalized to the MNI template (3 mm isotropic voxels) using the DARTEL toolbox in SPM8 (Ashburner, 2007). Combined transformations to the MNI template were then applied to each realigned EPI image, and those images were subsequently smoothed using a Gaussian kernel with an 8 mm full width at half maximum. After normalization of the EPI images, data from all voxels were linearly detrended and bandpass filtered (second-order Butterworth; 0.01–0.08 Hz) to minimize the effect of physiological artifacts on the resting-state signal. Subsequent functional connectivity analyses were confined to a mask of gray matter voxels from the segmented MNI template using custom-built tools.

### Seed Definition

Seed regions were generated using the MarsBar Matlab toolbox^2^. A 5 mm radius sphere was centered on a region of interest (ROI) in each subject’s spatially preprocessed data. Seeds were placed bilaterally in four ROIs: (1) area LC (LC), (2) caudate head (CH), (3) nucleus accumbens (NA), and (4) primary auditory cortex (A1), resulting in a total of eight seed ROIs. Seeds for LC and CH were anatomically defined individually by centering the seed over the region based on that subject’s anatomical T1-weighted MRI. The NA seeds were chosen from the anatomical location of the accumbens from the Wake Forest University (WFU) PickAtlas toolbox^3^ with the 5 mm sphere placed at the center of that location for every subject. For a particular A1 seed, a mask of the transverse temporal gyrus (TTG) was generated using the WFU PickAtlas. The seed region defined the center of mass. Functional connectivity estimates with the rest of the brain were computed separately for left and right seed ROIs.

### Functional Connectivity Analysis and Group Statistics

**FIGURE 1:**
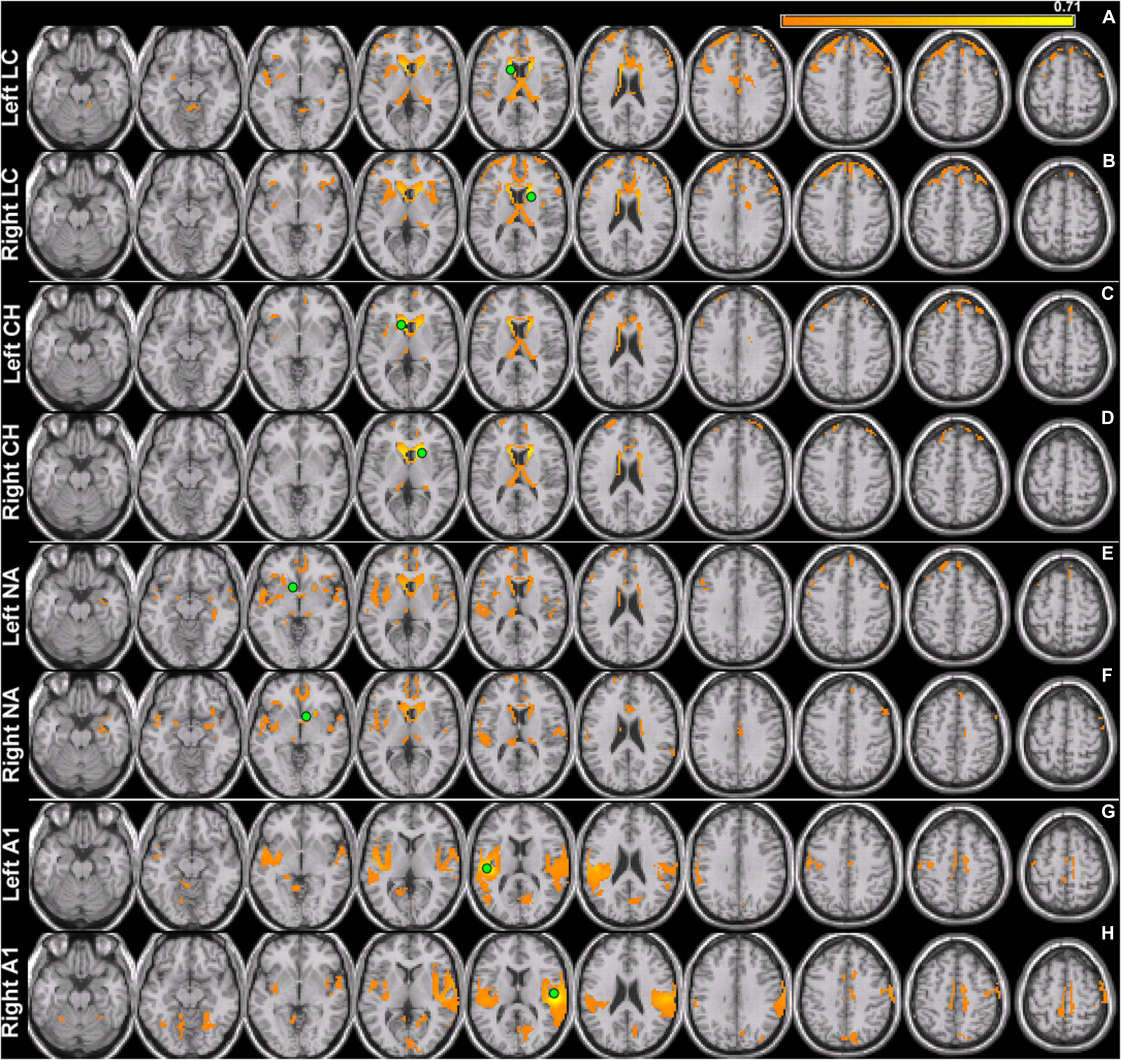
**Seed connectivity patterns in control subjects.** Seeds (green) are placed in the left and right hemispheres of area LC (LC), caudate head (CH), nucleus accumbens (NA), and primary auditory cortex (A1). Within-subject group averages in the control cohort for the eight seeds that are used to identify resting-state networks show unique connectivity patterns **(A–H)** for each pair. Significant functionally connected voxels include those underneath the marked seed (green circle). All maps are thresholded (80% maximum coherence value) and superimposed on horizontal slices (left-to-right, *z* = –22, –12, –2, 5, 15, 25, 32, 42, 52, 62) of a template brain using MRICro (http://www.mccauslandcenter.sc.edu/mricro/).

Functional connectivity between each ROI and the rest of the brain was computed using magnitude coherence (Coh) at low-frequency (<0.08Hz) oscillations of the BOLD signal. Magnitude coherence is a metric that estimates correlation in the frequency domain (see Brillinger, 2001; Muller et al., 2001; Sun et al., 2004; Hinkley et al., 2013). Following spatial and temporal preprocessing of EPI images, coherence estimates were calculated between the time series for each voxel in the seed with all remaining voxels in the brain, producing a single whole-brain coherence map for each seed voxel. Coherence values between each seed voxel with all other voxels in the brain were then Fisher *Z*-transformed and averaged across all seed voxels to yield a functional connectivity map for that ROI with all other regions in the brain (**Figure 1**). In order to normalize data for between group comparisons, each subject’s functional connectivity map was standardized by taking the Fisher *Z*-transformed average score for each voxel and computing a *Z*-score across all voxels transformed prior to within-group averaging as well as across-group second-level statistics. Voxelwise comparisons between groups (TIN vs. CON) were performed using analysis of covariance (ANCOVA) with group as a factor, with HL magnitude as a covariate. Corrections for multiple comparisons were performed using a cluster thresholding statistic on the ANCOVA results (*p* < 0.0075, *k* = 12 contiguous voxels).

## Results

### Connectivity Patterns in Controls

Seed connectivity patterns of those ROIs in control subjects provide reference information for comparisons. **Figure 1** shows significant resting-state functional connectivity patterns of the four ROIs (LC, CH, NA, and A1) in control subjects. Although unique connectivity patterns are evident for each seed, there is considerable overlap for area LC and CH. Left and right LC (**Figures 1A,B**): connections to dorsomedial prefrontal cortex (dmPFC) and the insula, and basal ganglia bilaterally. Medial and lateral variations in LC seed locations yield similar patterns of functional connectivity. Left and right CH (**Figures 1C,D**): also shows connections with dmPFC bilaterally and regions of the insula and neighboring structures of the basal ganglia. Left and right NA (**Figures 1E,F**) in the ventral striatum: connections with vmPFC, cerebellum, superior temporal lobe and posterior cingulate cortex bilaterally (lower middle rows). Left and right A1 (**Figures 1G,H**): connections to each other, cerebellum, medial prefrontal structures, including dmPFC and the supplementary motor area (SMA), as well as the cuneus in the occipital lobe.

### Tinnitus vs. Control Analyses: Subcortical Seeds

**FIGURE 2:**
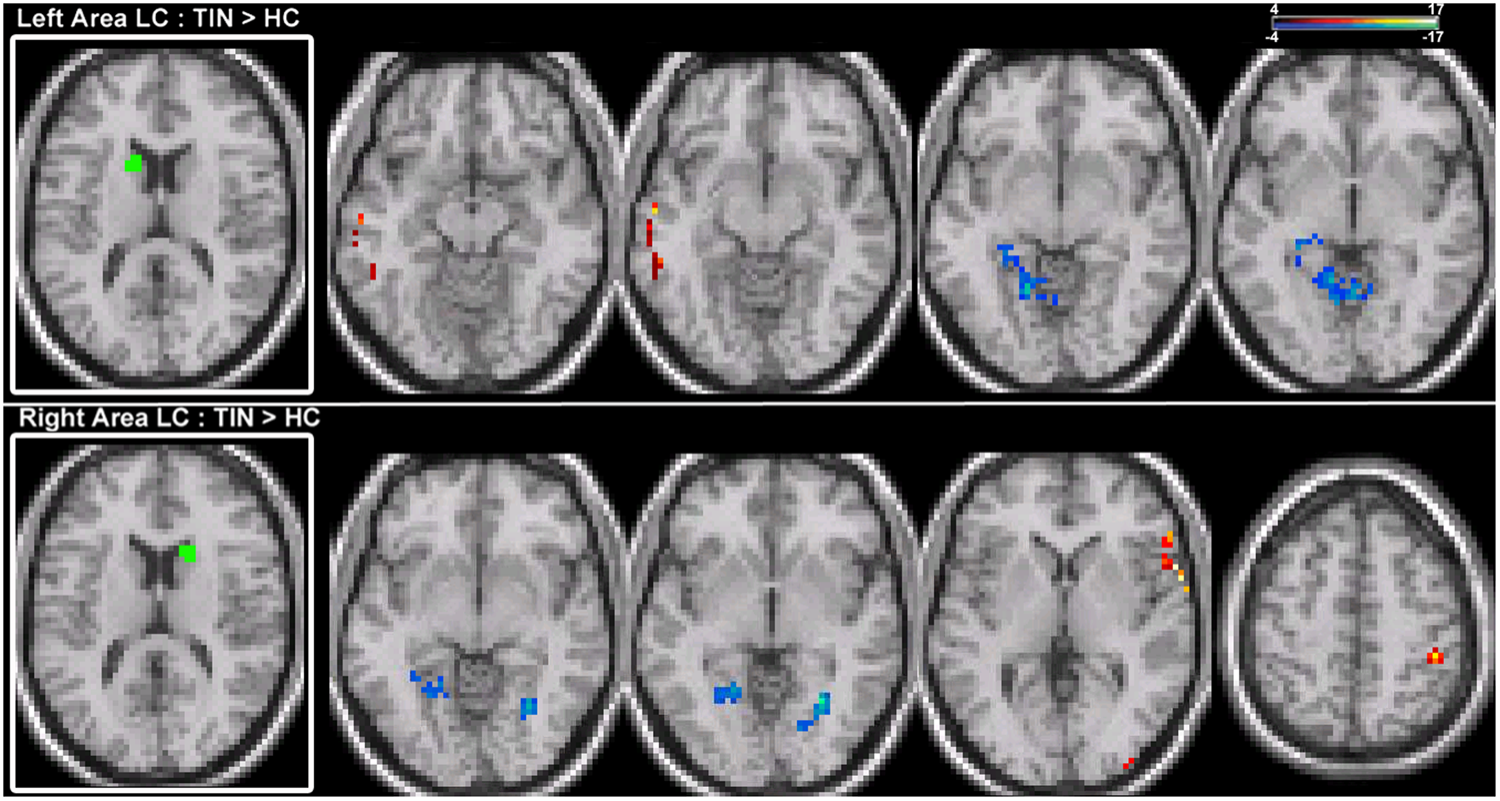
**Comparison of tinnitus vs. control: area LC seeds.** ANCOVA with hearing loss (HL) as a covariate (thresholded at 0.0075, *k* = 12). **(Top)** Left LC chronic tinnitus resting-state connectivity is increased (red) with auditory regions of the middle temporal gyrus (MTG) and decreased (blue) with lingual gyrus and cerebellum (left-to-right, *z* = –14, –13, –8, –6). **(Bottom)** Right LC chronic tinnitus resting-state connectivity is increased with auditory regions of the superior temporal gyrus (STG), middle occipital gyrus (MOG), and post-central gyrus (PoCG) and decreased with the lingual gyrus (left-to-right, *z* = –5, –2, 2, 52). All coordinates are in MNI space and functional overlays (color bar = *F*-value) performed using MRICro.

For left and right area LC, there are statistically significant increases in connectivity to the ipsilateral medial temporal gyrus (MTG) and superior temporal gyrus (STG) in the tinnitus cohort (**Figure 2**; **Table 2**). This increased functional connectivity is still significant after accounting for HL in the poorer ear (**Table 1**). Therefore, connectivity strength of area LC with auditory cortex is abnormally increased in chronic tinnitus. This relationship supports the hypothesis that striatal dysfunction in tinnitus may be the conduit for passage of auditory phantoms that reside in the central auditory system into perceptual awareness. Beyond increased auditory corticostriatal coherence, increased connections are also present between right area LC and the middle occipital gyrus (MOG) and post-central gyrus (PoCG; **Figure 2**; **Table 2**). Decreases in resting-state functional connectivity are identified in connections between both left and right LC and the lingual gyrus and left LC with the cerebellum (**Figure 2**; **Table 2**).

**Table 2:** Target region locations (labels for local maxima and *x, y, z* coordinates), *p*-value at the local maxima and cluster size (in voxels) from a group comparison between patients with chronic tinnitus and matched controls, with hearing loss level as a covariate.

Seed region	Targets : left hemisphere seed	*p*	Targets: right hemisphere seed	*p*	Target network
Area LC	↑ Left MTG (–58, –12, –13)	0.00740	↑ Right STG (62, 82)	0.00046	Auditory
	↓ Left lingual gyrus (–17, –59, –8)	0.00590	↓ Left lingual gyrus (–11, –58, –2) ↓ Right lingual gyrus (23, –63, –5) ↑Right MOG (36, –95, 5)	0.00450 0.00044 0.00680	Visual
	↓ Left culmen (–14, –49, –6)	0.00660			Cerebellar
			↑ Right PoCG (44, –39, 52)	0.00210	Parietal
Caudate Head			↓ Left culmen (–8, –59, –6)	0.00360	Cerebellar
	↑ Right putamen (20, 7, –11)	0.00126			Basal Ganglia
			↓ Right lingual gyrus (24, –65, –8)	0.00740	Visual
	↑ Right MFG (3, 45, 47)	0.00240	↑ Left SFG (–3, 38, 54)	0.00720	Dorsal Pre-frontal
	↑ Right cingulate (5, 17, 47)	0.00520			Default mode network (DMN)
	↑ Right IPL (41, –40, 53)	0.00076	↑ Right IPL (42, –38, 51)	0.00110	Parietal
Nucleus Accumbens	↓ Right STG (65, –24, –1)	0.00600	↑ Left MTG (–60, –26, –12)	0.00650	Auditory
			↑ Left SFG (–4, 52, –21)	0.00210	Orbital pre-frontal
	↓ Rght culmen (10, –55, 6)	0.00079	↑ LeftPoCb (–12, –76, –23)	0.00400	Cerebellar
	↓ Left lingual gyrus (–16, –55, 7) ↓ Right lingual gyrus (19, –69, 6)	0.00220 0.00130	↑ Left lingual (–25, –76, –12)	0.00120	Visual
	↓ Left IPL (–36, –44, 48)	0.00630	↑ Left I PL (–40, –38, 48)	0.00420	Parietal
Primary Auditory Cortex	↑ Right anterior STG (52, 14, –25)	0.00740			Temporal pole
	↑ Left MTG (–65, –34, –6)	0.00730	↑ Left MTG (–60, –34, –7)	0.00740	Auditory
	↑ Left SFG (–6, 56, –21)	0.00210	↑ Left SFG (–6, 55, –21)	0.00230	Orbital pre-frontal
	↑ Left PoCb (–11, –79, –21)	0.00650			Cerebellar
	↑ Right PHCG (27, 3, –13)	0.00210			Hippocampal
	↑ Left lingual gyrus (–29, –82, –11)	0.00330	↑ Right MOG (50, –70, –12)	0.00710	Visual
			↑ Right PoCG (64, –24, 18)	0.00730	Parietal

*Target regions for the four different seed regions (area LC, CH, NA, and A1) are shown for each hemisphere. Target regions are organized by the target network shown in the last column. SFG, superior frontal gyrus; IPL, inferior parietal lobe; MTG, middle temporal gyrus; MOG, middle occipital gyrus; PoCG, post-central gyrus; STG, superior temporal gyrus; MFG, middle frontal gyrus; PHCG, parahippocampal gyrus; PoCb, posterior cerebellum.*

**FIGURE 3:**
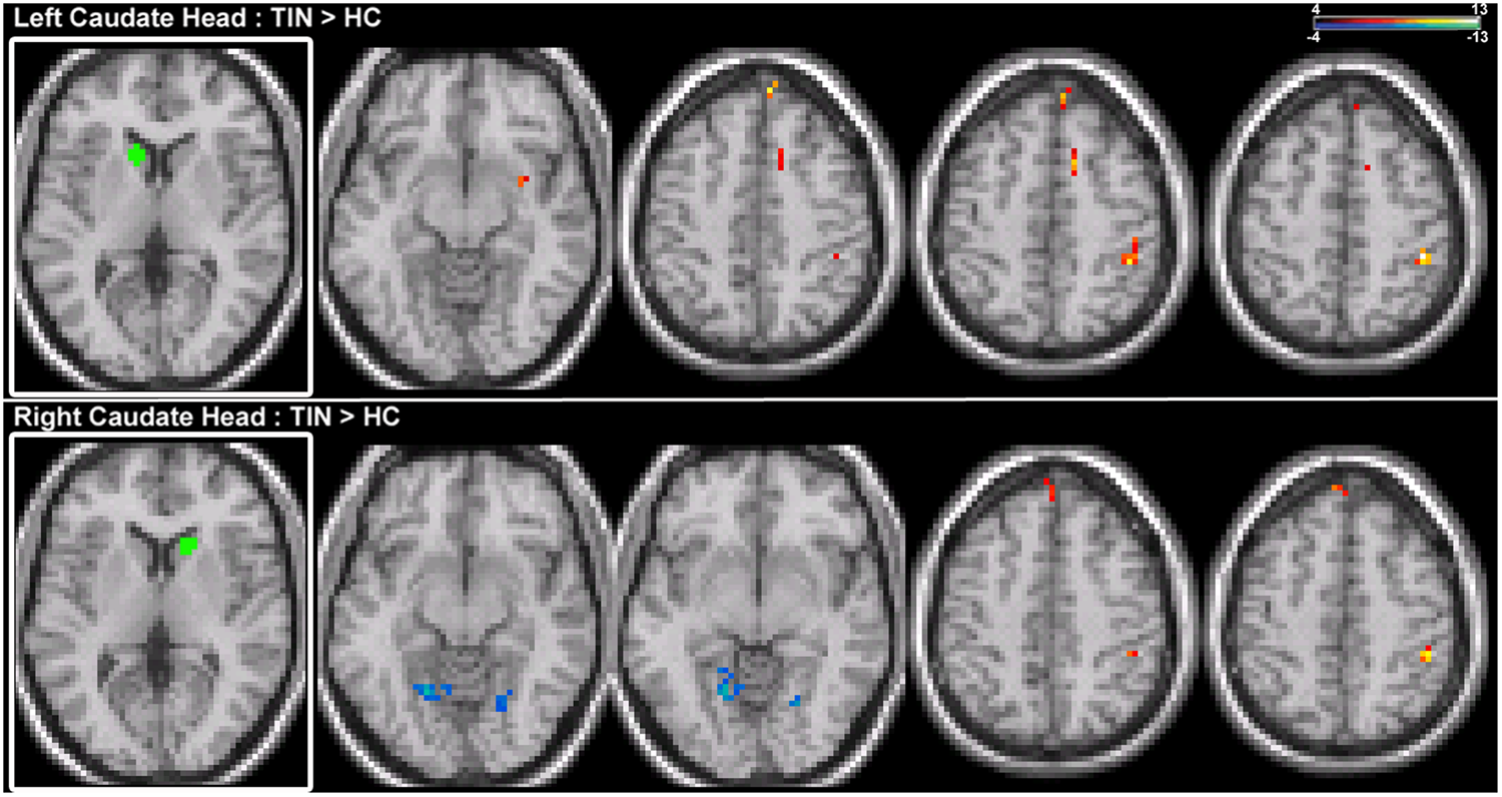
**Comparison of tinnitus vs. control: CH seeds. (Top)** Left CH chronic tinnitus resting-state connectivity is increased with the putamen, middle frontal gyrus (MFG), cingulate, and inferior parietal lobe (IPL) of the right hemisphere (left-to-right, *z* = –11, 46, 48, 53). **(Bottom)** Right CH chronic tinnitus resting-state connectivity is increased with the left dorsal superior frontal gyrus (SFG) and right IPL and decreased with the cerebellum and lingual gyrus (left-to-right, *z* = –8, –6, 51, 54). Conventions as in **Figure 2**.

Increases in resting-state coherence with the auditory system are not observed for the CH. Increased connections in the tinnitus cohort are observed for the left and right CH with dorsal pre-frontal cortex in the middle frontal gyrus (MFG) and superior frontal gyrus (SFG) and the right inferior parietal lobe (IPL; **Figure 3**; **Table 2**). Increased connections are also observed for left CH with the contralateral putamen and cingulate cortex (**Figure 3**; **Table 2**). Decreased connections in the tinnitus cohort are observed between right CH with the cerebellum and lingual gyrus (**Figure 3**; **Table 2**).

**FIGURE 4:**
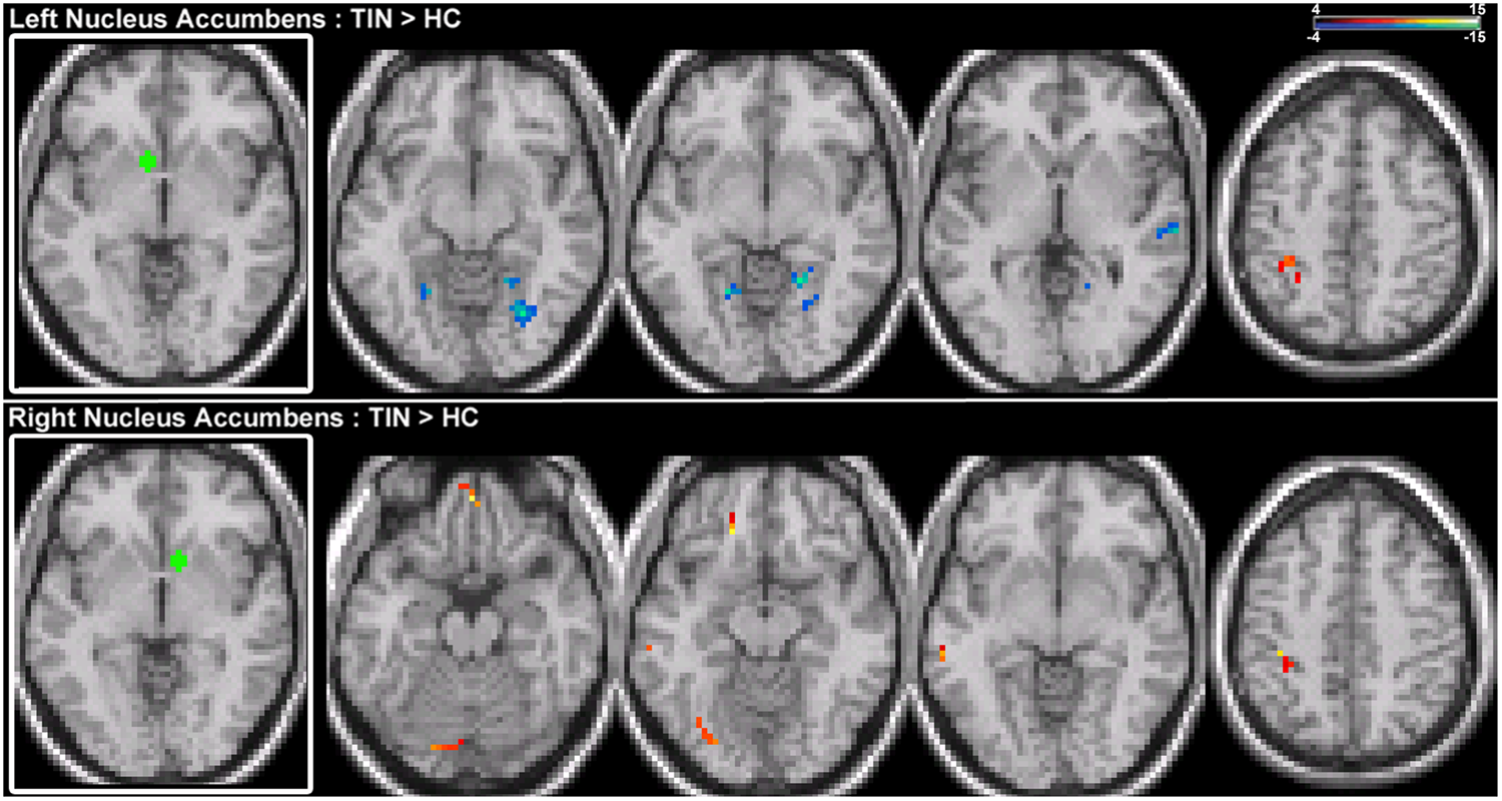
**Comparison of tinnitus vs. control: NA seeds. (Top)** Left NA chronic tinnitus resting-state connectivity is increased with the left IPL and decreased with the right STG, cerebellum, and lingual gyrus (left-to-right, *z* = –11, –6, –2, 48). **(Bottom)** Right NA chronic tinnitus resting-state connectivity is increased with the left MTG, ventral SFG, cerebellum, lingual gyrus, and IPL (left-to-right, *z* = –21, –12, –3, 48). Conventions as in **Figure 2**.

For connections of the NA, increased connectivity is observed between the right NA and left MTG, but not for the left NA (**Figure 4**; **Table 2**). Both regions show increased connectivity with the left IPL (**Figure 4**; **Table 2**). For left NA, decreased connections are observed with the right STG, cerebellum, and lingual gyrus (**Figure 4**; **Table 2**). For right NA, additional increased connections include left ventral SFG in orbitofrontal cortex, cerebellum, and lingual gyrus (**Figure 4**; **Table 2**). No decreased connections are detected for right NA.

### Tinnitus vs. Control Analyses: Cortical Seeds

**FIGURE 5:**
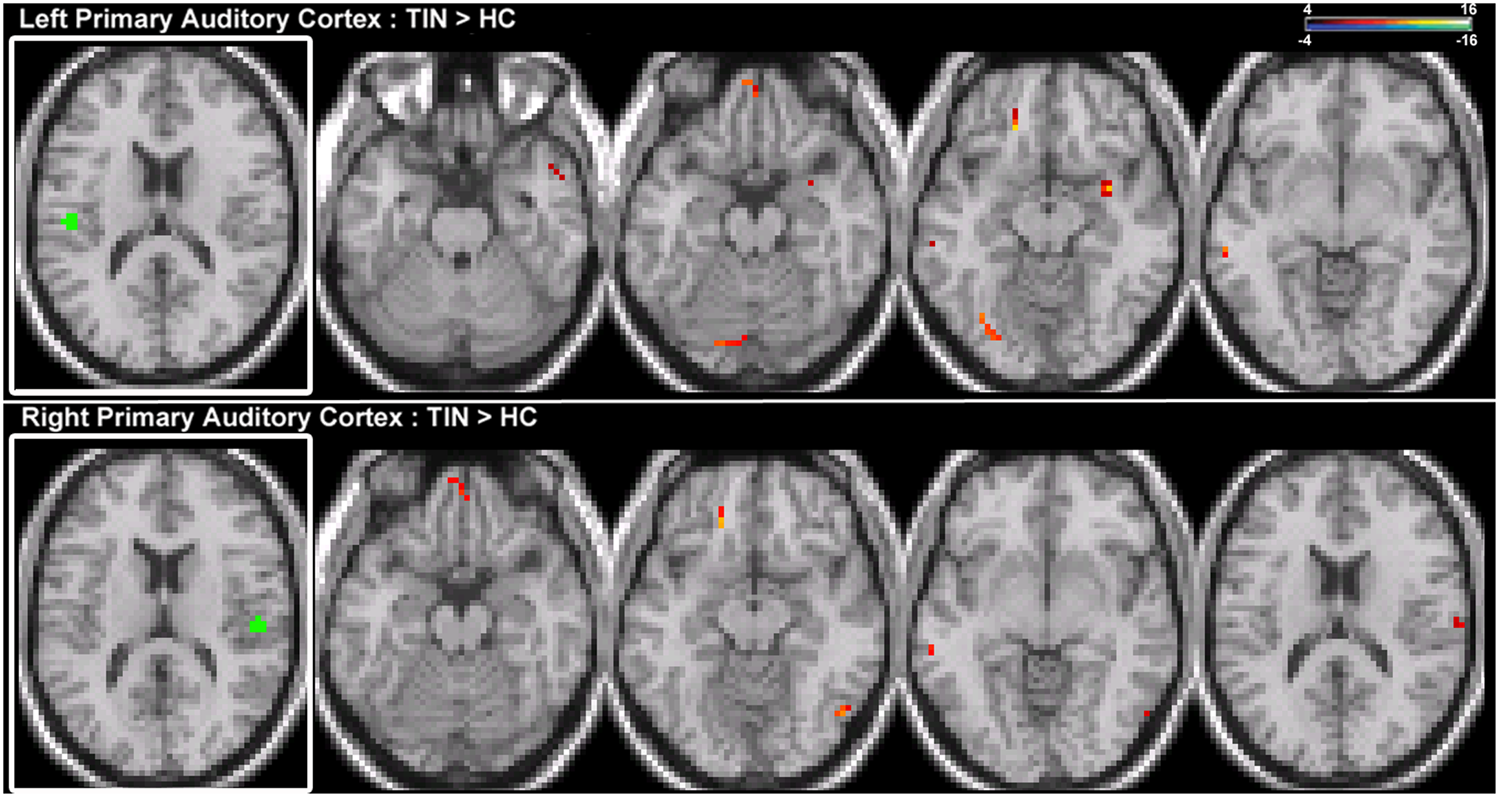
**Comparison of tinnitus vs. control: primary auditory cortex seeds. (Top)** Left primary auditory cortex (A1) chronic tinnitus resting-state connectivity is increased with the anterior STG, MTG, ventral SFG, cerebellum, parahippocampal gyrus (PHCG), and lingual gyrus (left-to-right, *z* = –21, –13, –11, –6). **(Bottom)** Right A1 chronic tinnitus resting-state connectivity is increased with the MTG, ventral SFG, MOG, and PoCG (left-to-right, *z* = –21, –18, –12, 17). Conventions as in **Figure 2**.

Seeds placed in left and right A1 exhibit alterations in resting-state functional connectivity. Increases in connectivity are observed between both seeds and left orbital pre-frontal cortex in the SFG and MTG (**Figure 5**; **Table 2**). Furthermore, there are increased connections between left A1 and the anterior STG, cerebellum, right parahippocampal gyrus (PHCG) and lingual gyrus. For right A1, increased connections are also identified with the right MOG and PoCG (**Figure 5**; **Table 2**). Collectively, the tinnitus cohort shows increased connectivity for both seeds between A1 and multiple networks encompassing orbital pre-frontal cortex, the PHCG, cerebellum, and visual cortex.

## Discussion

In chronic tinnitus patients adjusted for HL and compared with matched controls, the notable functional connectivity map finding is increased coherence between area LC and ipsilateral auditory cortical fields of the MTG and STG. This consistent, increased coherence is specific to dorsal striatal area LC and is distinct from patterns of connectivity at other sub-divisions of the basal ganglia, including the ventral striatum. Among unique connections of area LC, connections between the basal ganglia and auditory cortex are only discernible in the network for area LC in patients with chronic tinnitus, indicating specificity in the underlying neurobiology of auditory phantom percepts. Those findings provide further support for a basal ganglia-centric model and a potential platform to measure tinnitus objectively.

Increased connectivity of area LC with ipsilateral auditory cortex in chronic tinnitus may be altered by lesioning the dorsal striatum, where vascular insult to area LC causes tinnitus suppression to be more robustly expressed in the ipsilateral ear (Larson and Cheung, 2013). Whereas the dorsal striatum plays an important role in gating auditory phantom representations in auditory cortex for perceptual awareness, functional sub-divisions of the basal ganglia may play separate, but undefined roles in chronic tinnitus. Hyperconnectivity of area LC may be acting as a low-resistance conduit through which auditory phantoms represented in the central auditory system are gated into perceptual awareness. It should be noted that group differences in the connections of area LC to the MTG/STG remain significant following adjustment for HL. ANCOVA results account for HL magnitude by treating it as a covariate in the group analysis. This finding is congruent with the clinical observation that tinnitus severity as assessed by the THI is uncorrelated with the absolute and relative magnitude of HL in the poorer ear (Tsai et al., 2012).

Beyond abnormal basal ganglia connectivity, chronic tinnitus patients also have abnormal patterns of auditory cortical connectivity. A1 has increased coherence with the PHCG, cerebellum, and orbital pre-frontal cortex, a major hub of the default mode network (DMN; Greicius et al., 2003). While not directly related to the striatal gating model, it is possible that increased connectivity between A1 and subregions of the DMN (as well as CH with the DMN) may be related to introspection in this cohort, a function known to be modulated by the DMN (Fransson, 2005). Previous studies have shown that the strength of regional functional connectivity (global cross-correlations of the BOLD signal) for regions of the DMN are related to the amplitude of auditory phantom percepts (Ueyama et al., 2013), though this relationship between A1 and the DMN is not replicated in the current study.

Neuroanatomical tracer studies of connections between the striatum with surrounding cortical fields in the temporal lobe are remarkable for dorsal striatal connectivity to auditory cortex outside A1 (Reale and Imig, 1983; Selemon and Goldman-Rakic, 1985; Parent and Hazrati, 1995; Yeterian and Pandya, 1998). Besides A1, rostral auditory belt fields connect directly to the caudate in marmoset monkeys (de la Mothe et al., 2006). Similar patterns of connections have been identified in humans using non-invasive neuroimaging studies (Postuma and Dagher, 2006; Robinson et al., 2012). It follows that requisite corticostriatal neural circuitry is in place for dysfunctional striatal connectivity to enable perception of auditory phantoms. Altered auditory corticostriatal connectivity may drive change to a phantom percept network state where connectivity strengths of dense interconnections (Middleton and Strick, 2002) between the striatum and prefrontal cortical fields (dorsolateral, ventrolateral, medial, cingulate) are rebalanced. This notion is supported in the present study by our observations in the tinnitus cohort of increased connectivity with either medial pre-frontal, orbital pre-frontal, or cingulate cortex. However, the clinical correlates of those aberrant striatal–prefrontal connections in tinnitus remain to be defined. Voxelwise correlations with THI scores in the tinnitus cohort were insignificant when corrected for multiple comparisons. Future studies will need to investigate, in larger cohorts, relationships between tinnitus variables (such as distress, loudness) with patterns of connectivity to isolate functional substrates of pathologic networks. Functional analysis can be complemented with anatomical measurements. For example, volumetric analysis shows reduced gray matter in vmPFC where the magnitude of reduction is correlated with tinnitus loudness (Leaver et al., 2012).

This first study to evaluate directly connectivity patterns of striatum sub-divisions in chronic tinnitus validates the striatal gating model and confirms findings of prior resting-state EEG and fMRI studies (Vanneste et al., 2011; Maudoux et al., 2012a,b). Increases in functional connectivity with the PHCG are also observed in EEG studies of tinnitus, particularly in responders to transcranial stimulation (Vanneste et al., 2011). Similar to the patterns of resting-state functional connectivity we observe here, coherent oscillations between auditory cortex and the posterior cingulate are related to tinnitus symptoms (Vanneste and De Ridder, 2015). Studies using Independent Component Analysis on resting-state fMRI data have identified increased, aberrant connectivity of regions including the PHCG and parietal lobe (Maudoux et al., 2012b; Schmidt et al., 2013) and basal ganglia, and NA and cerebellum (Maudoux et al., 2012a,b). Interestingly, a recent study inducing tinnitus pharmacologically (Chen et al., 2015) reports increased connections between auditory cortex and the cerebellum and hippocampal gyrus, in concordance with the present study. Our finding of reduced connectivity in the lingual gyrus is consistent with the observations of Lehner et al. (2014), where reduced gray matter volume correlates with improvement in symptoms following TMS. Increased connections between the auditory network and dmPFC were reported in a small sample of tinnitus patients on a resting-state fMRI study (Kim et al., 2012), but other studies have failed to reproduce this finding (Schmidt et al., 2013; Davies et al., 2014). Heterogeneity among tinnitus cohorts may be contributing to network state variations.

There are several limitations to this study. Our sample size of 15 subjects in the chronic tinnitus cohort is relatively small. While our tinnitus and control cohorts are well matched for gender, age, and handedness, they are not matched for HL. HL is inhomogeneous in the poorer ear (**Table 1**). Tinnitus laterality is bilateral in 8 and unilateral in 7. This study is not sufficiently powered to ascertain whether tinnitus laterality is associated with distinct patterns of corticostriatal connectivity. Despite this heterogeneity, robust group differences manifest as increases in unilateral functional connectivity between area LC and auditory cortex in chronic tinnitus. Future studies on corticostriatal connectivity may address potential confounds by matching HL profiles of the tinnitus and control cohorts (as in Schmidt et al., 2013) or studying tinnitus patients with no HL at all (as in Chen et al., 2014). Larger cohorts can address the extent to which HL and tinnitus laterality impacts coherence in the BOLD signal between regions (Tibbetts et al., 2011; Schmidt et al., 2013), and reduce the likelihood of false positives. Although acquisition parameters in this study provide stability in the signal (Van Dijk et al., 2010) future studies examining longitudinal scale resting-state functional connectivity would require longer acquisition times (Birn et al., 2013). With those next steps, we can start to unravel how alterations in connectivity affect perceptual, attentional, and emotional aspects of tinnitus among subgroups.

In summary, the current work contributes to a growing body of literature examining corticostriatal interactions in tinnitus. A testable hypothesis of the striatal gating model of tinnitus has been assessed using resting-state fMRI. The physiologically based model derived from awake, interactive humans reporting on tinnitus modulation from direct electrical stimulation of the caudate nucleus predicts abnormal connectivity between area LC and auditory cortex. Results from this study have taken a step forward to validate the striatal gating model.

## Conflict of Interest Statement

The authors declare that the research was conducted in the absence of any commercial or financial relationships that could be construed as a potential conflict of interest.

## Footnotes

1 http://www.fil.ion.ucl.ac.uk/spm/software/spm8/

2 http://marsbar.sourceforge.net

3 fmri.wfubmc.edu/software/PickAtlas
